# Ictal vocalizations in the *Scn1a* ^+/−^ mouse model of Dravet syndrome

**DOI:** 10.1002/epi4.12715

**Published:** 2023-05-15

**Authors:** Lyndsey L. Anderson, Declan Everett‐Morgan, Stela P. Petkova, Jill L. Silverman, Jonathon C. Arnold

**Affiliations:** ^1^ Lambert Initiative for Cannabinoid Therapeutics The University of Sydney Camperdown New South Wales Australia; ^2^ Discipline of Pharmacology, School of Pharmacy Faculty of Medicine and Health, The University of Sydney Camperdown New South Wales Australia; ^3^ Brain and Mind Centre The University of Sydney Camperdown New South Wales Australia; ^4^ Department of Psychiatry and Behavioral Sciences, MIND Institute, School of Medicine University of California Davis California USA

**Keywords:** Dravet syndrome, epilepsy, seizures, ultrasonic, vocalizations

## Abstract

**Objective:**

Ictal vocalizations have shown diagnostic utility in epilepsy patients. Audio recordings of seizures have also been used for seizure detection. The present study aimed to determine whether generalized tonic–clonic seizures in the *Scn1a* ^+/−^ mouse model of Dravet syndrome are associated with either audible mouse squeaks or ultrasonic vocalizations.

**Methods:**

Acoustic recordings were captured from group‐housed *Scn1a* ^+/−^ mice undergoing video‐monitoring to quantify spontaneous seizure frequency. We generated audio clips (n = 129) during a generalized tonic–clonic seizure (GTCS) that included 30 seconds immediately prior to the GTCS (preictal) and 30 seconds following the conclusion of the seizure (postictal). Nonseizure clips (n = 129) were also exported from the acoustic recordings. A blinded reviewer manually reviewed the audio clips, and vocalizations were identified as either an audible (<20 kHz) mouse squeak or ultrasonic (>20 kHz).

**Results:**

Spontaneous GTCS in *Scn1a* ^+/−^ mice were associated with a significantly higher number of total vocalizations. The number of audible mouse squeaks was significantly greater with GTCS activity. Nearly all (98%) the seizure clips contained ultrasonic vocalizations, whereas ultrasonic vocalizations were present in only 57% of nonseizure clips. The ultrasonic vocalizations emitted in the seizure clips were at a significantly higher frequency and were nearly twice as long in duration as those emitted in the nonseizure clips. Audible mouse squeaks were primarily emitted during the preictal phase. The greatest number of ultrasonic vocalizations was detected during the ictal phase.

**Significance:**

Our study shows that ictal vocalizations are exhibited by *Scn1a* ^+/−^ mice. Quantitative audio analysis could be developed as a seizure detection tool for the *Scn1a* ^+/−^ mouse model of Dravet syndrome.

**Key points**

- Audible mouse squeaks precede spontaneous seizures in *Scn1a* ^+/−^ mice.
- Increased number of ultrasonic vocalizations exhibited by *Scn1a* ^+/−^ mice in association with generalized tonic–clonic seizure activity.
- Ultrasonic vocalizations primarily occur during the ictal phase.

## 1 INTRODUCTION

Devices used to detect and alert for seizures have become increasingly popular with epilepsy patients and their caretakers. Carers alerted to seizure activity are able to intervene, which could reduce the risk of sudden unexpected death in epilepsy (SUDEP). Noninvasive seizure detection devices include movement detectors and autonomic change detectors that measure heart rate, respiration, or electrodermal response.^1^ Audio‐based seizure detection is also being explored since ictal vocalizations have been described in many epilepsy patients.^2^, ^3^, ^4^ Given the laborious nature of seizure quantification in rodent epilepsy models, audio‐based seizure detection might help in the development of automated seizure detection technologies.

Audible sounds are characteristic of several seizure types. The guttural, expiratory sound known as the “ictal cry” is a stereotypical feature of generalized tonic–clonic seizure (GTCS).^4^, ^5^ During the tonic phase, contraction of the axial and abdominal muscles causes the diaphragm to slowly force air through the vocal cords giving rise to the ictal cry. Ictal vocalizations, which include both intelligible speech and nonspeech sounds, are frequently observed during focal seizures.^6^, ^7^, ^8^, ^9^

The prevalence of ictal vocalizations may provide diagnostic utility in epilepsy patients, so we aimed to determine whether GTCS in the *Scn1a* ^+/−^ mouse model of Dravet syndrome is also associated with vocalizations. Audible vocalizations have been observed at the onset of seizure behavior in rats.^10^, ^11^ In our laboratory, we have observed that audible mouse squeaks commonly precede GTCS activity and noted that during seizure activity, a mouse's mouth remains open despite no audible sound being heard. Communicative behavior of rodents is predominantly in the form of ultrasonic vocalizations, which are aerodynamic whistles occurring between 20–120 kHz.^12^ Rodent ultrasonic vocalizations have been implicated in parent‐offspring interactions, mating behavior, social interactions, emotional status and to warn of a threat.^13^, ^14^, ^15^, ^16^, ^17^, ^18^, ^19^ A recent study showed that rats emit ultrasonic vocalizations during induced seizures.^20^ Thus, we hypothesized that *Scn1a* ^+/−^ mice emit ultrasonic vocalizations during a seizure. In the current study, we investigated whether ictal vocalizations either as audible mouse squeaks or ultrasonic vocalizations are characteristics of *Scn1a* ^+/−^ mice.

## 2 METHODS

### 2.1 Animals

All animal care and procedures were approved by the University of Sydney Animal Ethics Committee in accordance with the Australian Code of Practice for the Care and Use of Animals for Scientific Purposes. Mice heterozygous for *Scn1a* (*Scn1a* ^+/−^) were purchased from The Jackson Laboratory (stock 37107‐JAX; Bar Harbor, USA). *Scn1a* ^+/−^ mice were maintained as a congenic line on the 129S6/SvEvTac background and were bred with C57BL/6J mice to generate experimental mice on an F1 genetic background. The *Scn1a* genotype was determined as previously described.^21^ Mice were group‐housed in specific pathogen‐free mouse facilities under standard conditions (12‐hour light/12‐hour dark cycle) with ad libitum access to food and water.

### 2.2 Vocalization recordings

Vocalizations were recorded using an UltraSoundGate condenser microphone CM16/CMPA (Avisoft Bioacoustics; Berlin, GER) connected via an UltraSoundGate 416H audio device (Avisoft Bioacoustics) to a personal computer. Acoustic data were recorded as a WAV file with a sampling rate of 250 kHz in 16‐bit format by Avisoft RECORDER USGH (version 4.2.30). Specific recording conditions for each experiment are described below. Avisoft SASLab Pro (version 5.2.15) with a 256 fast Fourier transform was used to generate spectrograms. An investigator blinded to the experimental condition identified calls manually. Calls were categorized as either ultrasonic (>20 kHz) or audible (<20 kHz) mouse squeaks. A separate investigator blinded to the experimental condition listened to the audio files to confirm mouse squeaks. The false‐positive rate for audible mouse squeaks called from the spectrogram was 2.5%.

### 2.3 Spontaneous seizures

Male and female *Scn1a* ^+/−^ mice were exposed to a single hyperthermia‐induced seizure event at P18 as described previously.^22^ At P19, mice were then housed in a recording chamber (28 × 28 × 36 cm) in groups of 2–3 with at least one of each sex. Eleven cages of 28 *Scn1a* ^+/−^ mice were recorded from 12:00 on P19 through 24:00 on P21. Overhead video was captured using a Day/Night camera (Samsung SCB5003) equipped with an infrared lens (Tamron 13FG04IRSQ). Microphones for acoustic recordings were placed 20 cm above the floor of the recording chambers. During recording, mice had access to food and water ad libitum. Spontaneous generalized tonic–clonic seizures (GTCS) were captured by continuous video recording over 60 hours (12:00 on P19 through 24:00 on P21). Previous studies in *Scn1a* ^+/−^ mice showed a perfect correlation (*κ* = 1.0) between behavioral and electroencephalographic GTCS captured by video and EEG, respectively.^23^ Digital videos captured during the session were analyzed offline and a total of 129 spontaneous seizures were identified. The severity of each GTCS was scored for progression to full hindlimb extension (hindlimbs at 180° angle to the torso), the most severe stage of GTCS. Seizure and nonseizure clips were exported from the acoustic recordings. Seizure clips (n = 129) included 30 seconds immediately prior to the GTCS (preictal) and 30 seconds following the conclusion of the seizure (postictal). Nonseizure clips (n = 129) were at least 10 minutes away from a seizure clip. Acoustic recordings were also captured from an empty cage (n = 15) adjacent to a recording cage to account for background vocalizations since no sound attenuation was used. A total of 1083 minutes (57 minutes, empty cage; 514 minutes, nonseizure; 512 minutes, seizure) of acoustic data were analyzed.

Statistical comparisons were made in GraphPad Prism 8.2 (La Jolla, USA) and *P* < 0.05 was considered statistically significant. The Kruskal–Wallis test followed by the Dunn's post hoc test was used to compare total vocalizations across groups. The Mann–Whitney test was used to compare parameters between nonseizure and seizure clips. Repeated measures of the Friedman's test followed by the Dunn's post hoc test were used to compare epochs (preictal, ictal, and postictal) in seizure clips.

## 3 RESULTS

### 3.1 Spontaneous seizures associated with greater vocalizations

*Scn1a* ^+/−^ mice commonly squeak during the pre‐ and early ictal phases of a GTCS. We hypothesized that these audible mouse squeaks were unique to seizure activity. Thus, we collected acoustic recordings of *Scn1a* ^+/−^ mice undergoing video‐monitoring to quantify spontaneous GTCS frequency. Video and acoustic recordings were taken from 28 *Scn1a* ^+/−^ mice group‐housed in eleven cages. Video (660 hours) was scored and 129 total spontaneous GTCS were identified. Seizure clips (n = 129) that encompassed 30 seconds immediately prior to the GTCS (preictal) and 30 seconds following the conclusion of the seizure (postictal) were exported from the acoustic recordings. The nonseizure clips (n = 129) were randomly exported from the acoustic recordings and were taken at least 10 minutes away from a documented seizure. Acoustic recordings were also captured from an empty cage (n = 15) adjacent to a recording cage to account for background vocalizations since no sound attenuation was used. A total of 1083 minutes (57 minutes, empty cage; 514 minutes, nonseizure; 512 minutes, seizure) of acoustic data were analyzed for both audible mouse squeaks and ultrasonic vocalizations. In the spectrogram, mouse squeaks appear as high‐intensity, sustained low‐frequency (approximately 7 kHz) linear structured calls, whereas ultrasonic vocalizations appeared as high‐intensity bands above 20 kHz (Figure S1).

**FIGURE 1:**
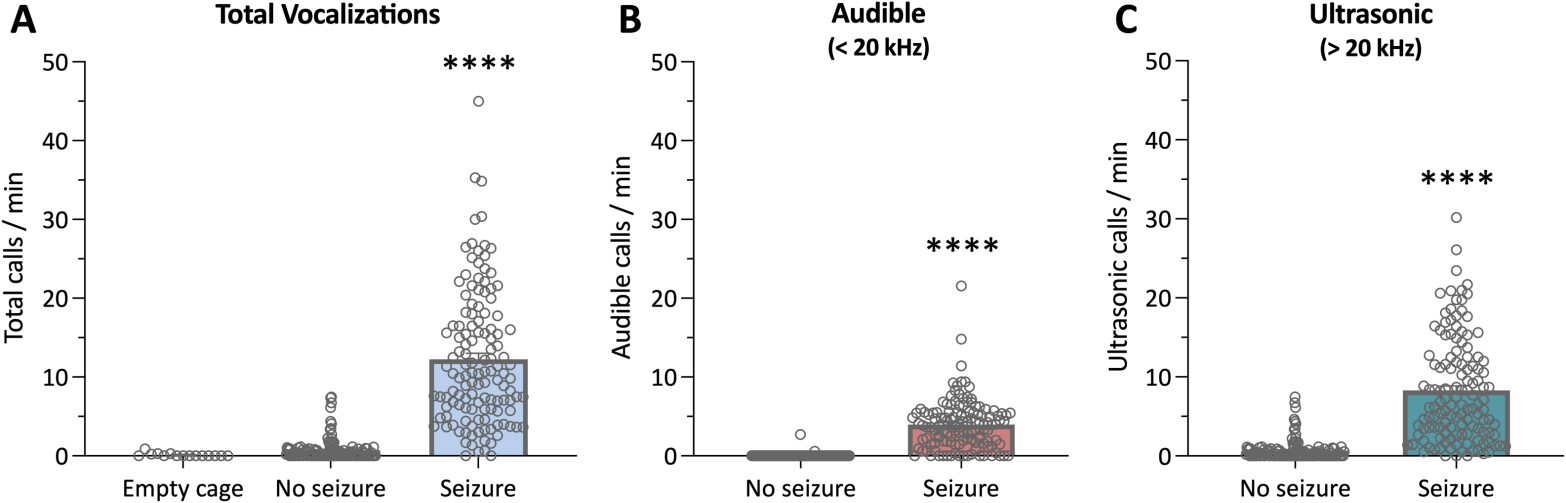
Vocalizations associated with spontaneous seizures in *Scn1a* ^+/−^ mice. (A) Total number of vocalizations emitted in individual recordings of group‐housed *Scn1a* ^+/−^ mice. Total calls included those in both the audible and ultrasonic ranges in acoustic recordings that did and did not contain a seizure. No sound attenuation was used so the empty cage accounts for background vocalizations. A significantly greater number of vocalizations were present in recordings that contained a generalized tonic–clonic seizure. Error bars represent SEM, with n = 15 (empty cage) and n = 129 per nonseizure and seizure group (****P < 0.0001; Kruskal–Wallis test followed by Dunn's post hoc). (B) Audible mouse squeaks emitted in individual recordings of group‐housed *Scn1a* ^+/−^ mice. Recordings that contained a generalized tonic–clonic seizure had significantly more mouse squeaks than recordings during a seizure‐free period. Error bars represent SEM, with n = 129 per group (****P < 0.0001; Mann–Whitney test). (C) Ultrasonic vocalizations (>20 kHz) emitted in individual recordings of group‐housed *Scn1a* ^+/−^ mice. A significantly greater number of ultrasonic vocalizations were present in the recordings that contained a generalized tonic–clonic seizure. Error bars represent SEM, with n = 129 per group (****P < 0.0001; Mann–Whitney test).

Spontaneous seizures in *Scn1a* ^+/−^ mice were associated with a high number of vocalizations (Figure 1A). In the seizure clips, a significantly greater number of vocalizations were observed in the seizure clips compared with both the empty cage (*P* < 0.0001) and nonseizure (*P* < 0.0001) clips. Total vocalizations were then separated into audible or ultrasonic groups and compared between the nonseizure and seizure clips. A significantly greater number of audible squeaks were recorded in the seizure clips (*P* < 0.0001). In fact, barely any of the nonseizure clips contained audible calls (2 of 129 clips), whereas nearly all of the seizure clips (114 of 129 clips) contained at least one audible mouse squeak (Figure 1B). The number of ultrasonic vocalizations in the seizure clips was also significantly greater than those of the nonseizure clips (*P* < 0.0001). Only three of the seizure clips did not contain an ultrasonic vocalization (126 of 129 clips contain ultrasonic vocalizations), two of which did not include an audible mouse squeak either (Figure 1C). By comparison, only 57% of nonseizure clips contained an ultrasonic vocalization (73 of 129 clips contain ultrasonic vocalizations).

**FIGURE 2:**
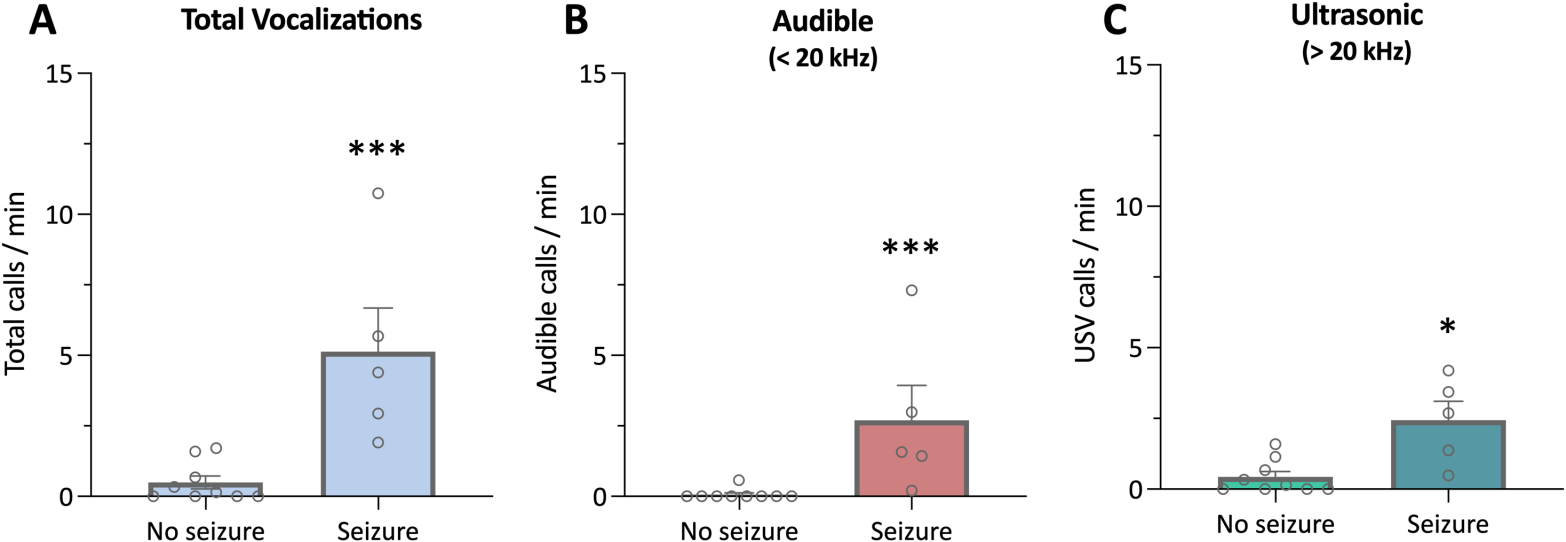
Increased vocalizations of individually‐housed *Scn1a* ^+/−^ mice during a seizure. (A) Total number of vocalizations emitted in recordings of *Scn1a* ^+/−^ mice singly‐housed. Total calls included those in both the audible and ultrasonic ranges in acoustic recordings that did and did not contain a seizure. *Scn1a* ^+/−^ mice vocalized significantly more during a seizure event. Error bars represent SEM, with n = 5–9 per group (***P < 0.005; Mann–Whitney test). (B) Audible mouse squeaks emitted by individual *Scn1a* ^+/−^ mice. Recordings that contained a generalized tonic–clonic seizure had significantly more mouse squeaks than those recorded during a seizure‐free period. Error bars represent SEM, with n = 5–9 per group (***P < 0.005; Mann–Whitney test). (C) Ultrasonic vocalizations (> 20 kHz) emitted by individual *Scn1a* ^+/−^ mice. A significantly greater number of ultrasonic vocalizations were present in the recordings that contained a generalized tonic–clonic seizure. Error bars represent SEM, with n = 5–9 per group (*P < 0.05; Mann–Whitney test).

As the mice are group‐housed, we are unable to say whether the seizing mouse or a cage‐mate is responsible for the vocalizations. However, *Scn1a* ^+/−^ mice have poor survival so there were several clips where only a single mouse remained in the cage. A significantly greater number of both audible (*P* = 0.001) and ultrasonic (*P* = 0.0105) vocalizations were detected in the seizure clips compared with the nonseizure clips of singly‐housed mice (Figure 2). While this does not discount that the cage‐mates could also vocalize during a seizure event, it does confirm that a seizing mouse emits vocalizations.

### 3.2 Increased ultrasonic vocalization frequency and duration associated with spontaneous seizures

**FIGURE 3:**
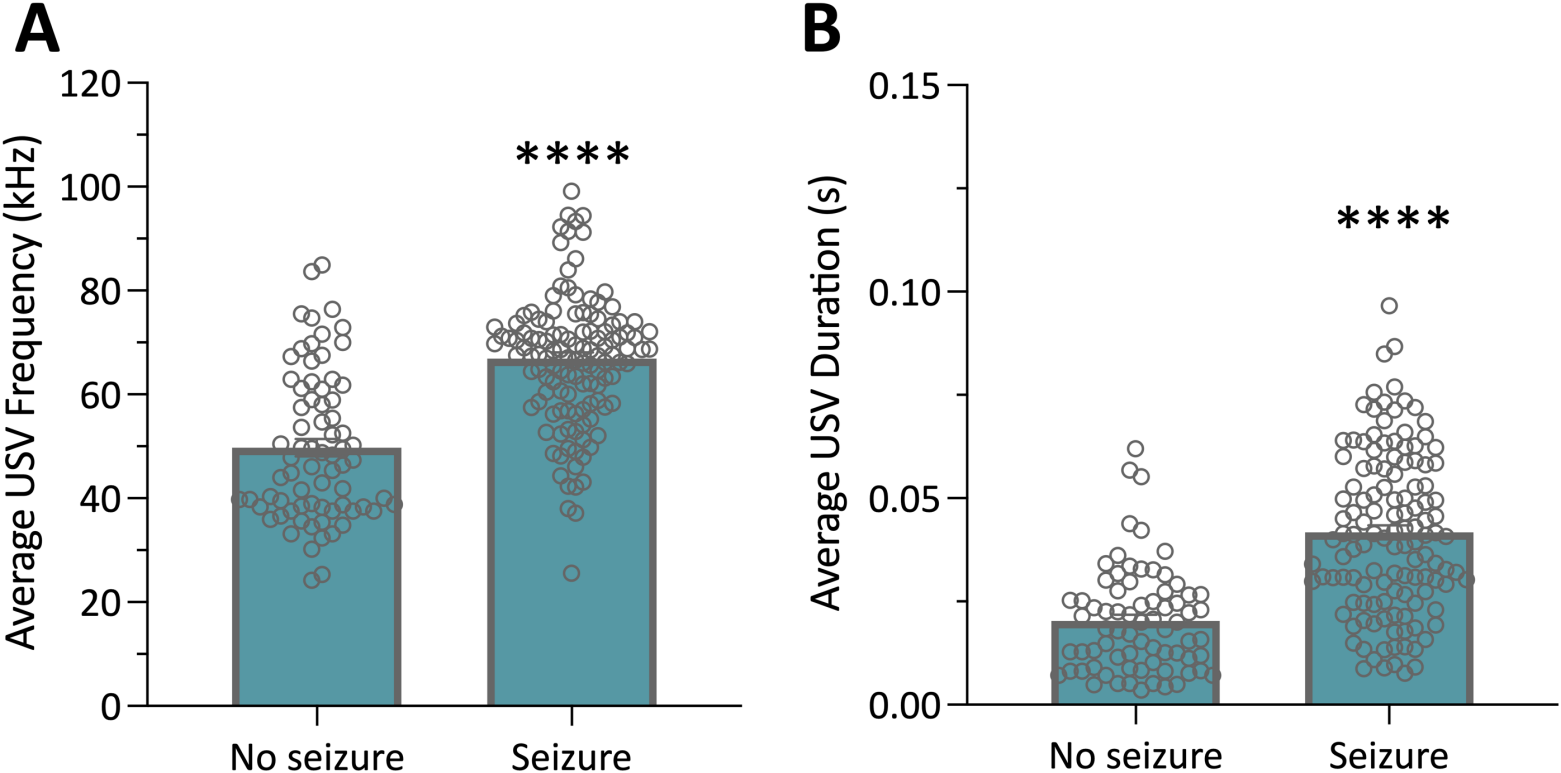
Spontaneous seizures in *Scn1a* ^+/−^ mice result in an increased frequency and duration of ultrasonic vocalizations. (A) Average frequency of ultrasonic vocalizations emitted in individual recordings of group‐housed *Scn1a* ^+/−^ mice. Ultrasonic vocalization frequency is significantly increased in recordings with a generalized tonic–clonic seizure. Error bars represent SEM, with n = 73–126 per group (****P < 0.0001; Mann–Whitney test). (B) Average duration of ultrasonic vocalizations emitted in individual recordings of group‐housed *Scn1a* ^+/−^ mice. Duration of ultrasonic vocalizations was significantly longer in the recordings that contained a generalized tonic–clonic seizure. Error bars represent SEM, with n = 73–126 per group (****P < 0.0001; Mann–Whitney test).

Acoustic recordings of *Scn1a* ^+/−^ mice experiencing a spontaneous GTCS contained a significantly greater number of ultrasonic vocalizations than recordings during a seizure‐free period. We next compared the parameters of the vocalizations to determine whether they also differed between groups. First, we examined the average frequency of the ultrasonic vocalizations (Figure 3A). The ultrasonic vocalizations in the seizure clips had an average frequency of 66.9 ± 1.2 kHz, which is significantly higher than the average frequency of 49.7 ± 1.7 kHz in the nonseizure clips (*P* < 0.0001). Next, the durations of the ultrasonic vocalizations were compared (Figure 3B). The average ultrasonic vocalization duration in the seizure clips (42 ± 2 msec) was twice as long as those in the nonseizure cohort (20 ± 2 msec).

### 3.3 Ultrasonic vocalizations increased during the seizure

**FIGURE 4:**
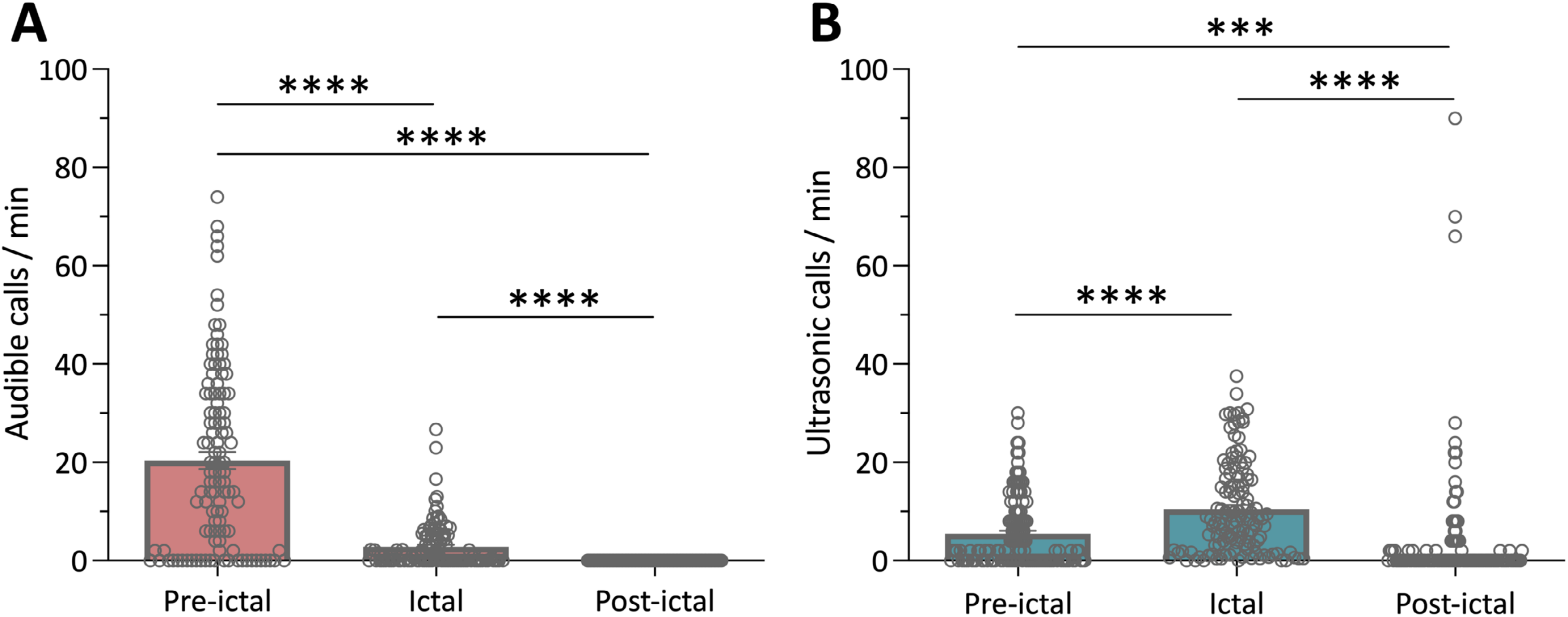
Vocalizations correspond phase of seizure in *Scn1a* ^+/−^ mice. (A) Audible mouse squeaks emitted in individual recordings of seizure events by group‐housed *Scn1a* ^+/−^ mice. Mouse squeaks were separated by seizure phase and occurred most often in the preictal stage. Error bars represent SEM, with n = 114 repeated measures (****P < 0.0001; Friedman's test followed by Dunn's post hoc). (B) Ultrasonic vocalizations emitted in individual recordings of seizure events by group‐housed *Scn1a* ^+/−^ mice separated by seizure phase. A significantly greater number of ultrasonic vocalizations occurred during the ictal phase. Error bars represent SEM, with n = 126 repeated measures (***P < 0.001, ****P < 0.0001; Friedman's test followed by Dunn's post hoc).

Vocalizations in *Scn1a* ^+/−^ mice significantly increased in association with a seizure and a common observation is that *Scn1a* ^+/−^ mice squeak prior to a GTCS. Thus, we aimed to determine whether vocalizations are affected by the phase of the seizure. Consistent with previous observations, audible mouse squeaks were most prevalent in the preictal phase (Figure 4A). There were significantly more audible calls in the preictal phase than in either the ictal (*P* < 0.0001) or postictal (*P* < 0.0001) phases. The ictal phase contained a significantly greater number of audible calls than the postictal phase, where no mouse squeaks were detected (*P* < 0.0001).

Ultrasonic vocalizations in relation to the seizure phase were examined next. Interestingly, the greatest number of ultrasonic vocalizations was detected during the ictal phase (Figure 4B). There were significantly more ultrasonic vocalizations in the ictal phase compared with either the preictal (*P* < 0.0001) or postictal (*P* < 0.0001) phases. There were also a significantly greater number of ultrasonic vocalizations in the preictal compared with postictal phase (*P* = 0.0008).

**FIGURE 5:**
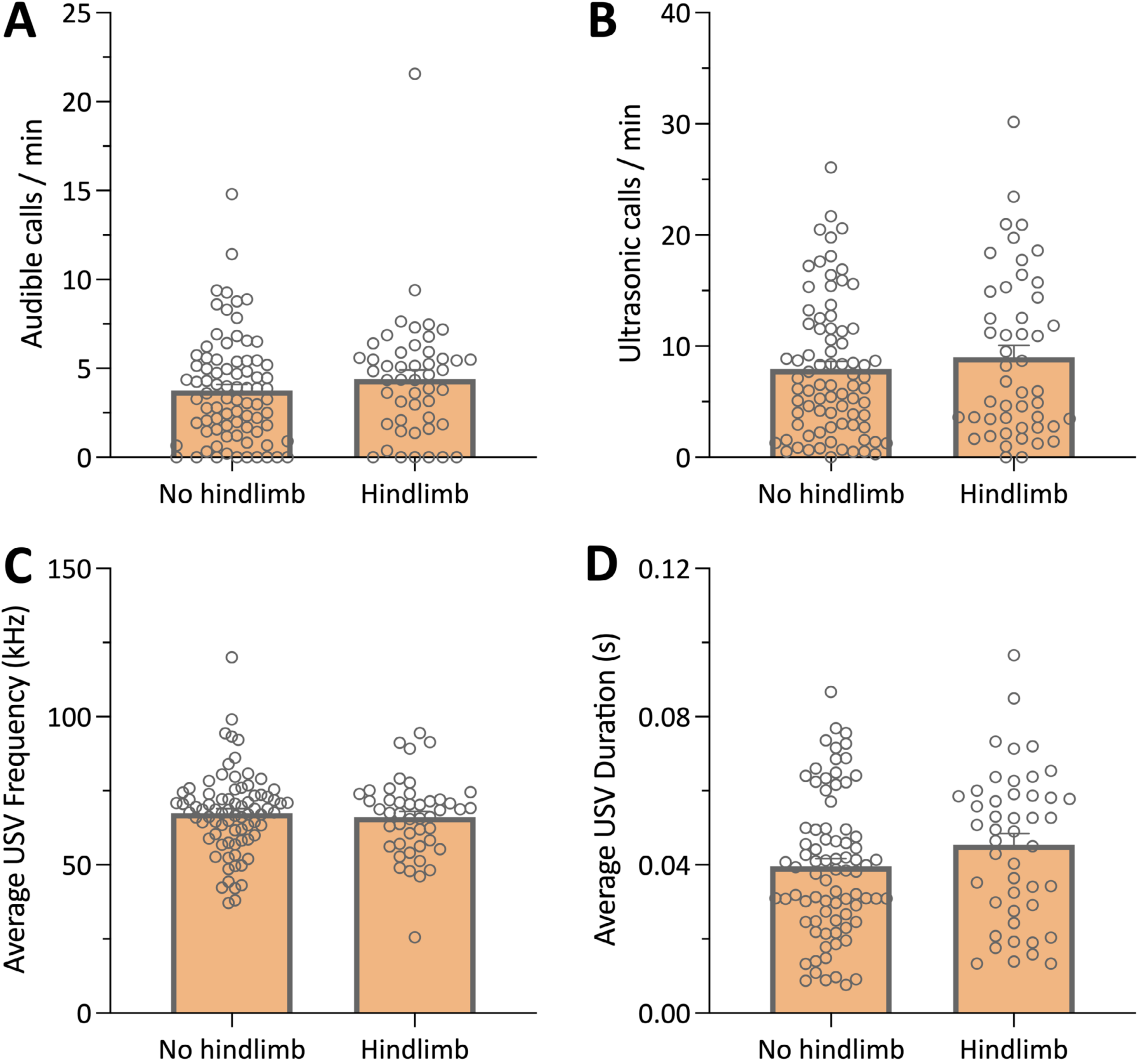
Seizure severity has no effect on vocalizations in *Scn1a* ^+/−^ mice. (A) Audible mouse squeaks or (B) ultrasonic vocalizations emitted by group‐housed *Scn1a* ^+/−^ mice with respect to generalized tonic–clonic seizure severity. Seizure severity did not affect the number of audible mouse squeaks or ultrasonic vocalizations. Error bars represent SEM, with n = 80 and 49 in the no hindlimb and hindlimb groups, respectively (Mann–Whitney test). (C) Average frequency and (D) duration of ultrasonic vocalizations emitted in individual recordings of group‐housed *Scn1a* ^+/−^ mice. Seizure severity did not affect the frequency or duration of ultrasonic vocalizations. Error bars represent SEM, with n = 80 and 46 in the no hindlimb and hindlimb groups, respectively (Mann–Whitney test).

Lastly, we examined whether ultrasonic vocalizations were affected by seizure severity. Each GTCS was scored for progression to full hindlimb extension, the most severe seizure stage. Seizure severity had no effect on ultrasonic vocalization number, frequency, or duration (Figure 5).

## 4 DISCUSSION

In the present study, we investigated whether the phenotype of the *Scn1a* ^+/−^ mouse model of Dravet syndrome includes ictal vocalizations. Analogous to the ictal cry associated with GTCS in epilepsy patients, an “ictal squeak” was observed in *Scn1a* ^+/−^ mice. Audible mouse squeaks were present in 88% of the GTCS recordings. Taking ultrasonic vocalizations into account, however, showed that 98% of the GTCS clips contained ictal vocalizations (audible mouse squeaks and/or ultrasonic vocalizations). We also found that the frequency and duration of ultrasonic vocalizations in association with GTCS were greater than those present in nonseizure control clips. Our presumption is that the vocalizations are emitted from the seizing mouse; however, it cannot be ruled out that some of the vocalizations could be attributed to the nonseizing cage‐mates since the mice were group‐housed. Since *Scn1a* ^+/−^ mice have poor survival, we did capture several clips where only a single mouse remained in the cage. In these instances, a significantly greater number of vocalizations were detected in the seizure clips compared with the nonseizure clips, supporting our presumption. Although sufficiently powered to detect a difference between nonseizure and seizure clips, our sample size of GTCS from singly‐housed mice was small, so future work will need to expand the dataset of individual *Scn1a* ^+/−^ mice. Nonetheless, regardless of whether the vocalizations are credited to the seizing mouse or nonseizing cage‐mates, ictal vocalizations are observed in *Scn1a* ^+/−^ mice. Notably, a recent study showed that rats emit ultrasonic vocalizations during seizures induced by pentylenetetrazole (PTZ).^20^ Our results extend on this recent finding by showing that spontaneous seizures are also associated with ultrasonic vocalizations. Collectively, these data suggest that quantitative audio analysis could be developed as a seizure detection tool for *Scn1a* ^+/−^ mice.

Quantification of spontaneous GTCS in the *Scn1a* ^+/−^ mouse model of Dravet syndrome currently relies on manual review of video and/or video‐EEG recordings.^22^, ^23^, ^24^, ^25^ Since monitoring tends to be across several days, quantifying GTCS frequency is time‐consuming and labor‐intensive. An audio‐based seizure detection device with machine‐learning algorithms to detect audible mouse squeaks and discern features of ultrasonic vocalizations could potentially allow for the quantification of GTCS frequency to be automated. Seizure frequency is often quantified in *Scn1a* ^+/−^ mice to assess the anticonvulsant efficacy of a novel treatment. Therefore, drug treatments, including novel chemical entities, would have to show no effect on vocalization ability before audio‐based seizure detection could be employed in such drug discovery programs.

Sensitivity and false alarm rates are important performance measures for seizure detection devices.^26^ Currently available automated seizure detection devices have sensitivity rates of 86–95% for tonic–clonic seizures in epilepsy patients.^27^, ^28^, ^29^ Focusing on audible mouse squeaks only, our experimenter performed with a sensitivity of 88%. Our data showed 2 false‐positive audible mouse squeaks in 129 nonseizure clips (514 minutes). Calculating a false alarm rate by converting this to a 24 hours period, our false alarm rate would be 5.6/day. This false alarm rate is substantially higher than that of commercially available seizure detection devices used in human epilepsy patients, which range from 0.2 to 0.67/day.^27^, ^28^, ^29^ When ultrasonic vocalizations are included in the ictal vocalization, the sensitivity improves to 98%, but the false alarm rate is worse (73 of the 129 nonseizure clips contained vocalizations). However, machine‐learning algorithms that detect not only the presence of vocalizations but also account for the frequency and duration of ultrasonic vocalizations have the potential to improve the false alarm rate.

A reliable automated program to analyze the vocalizations would need to first be established before audio‐based seizure detection could replace video or video‐EEG recordings. Here, vocalizations were collected and analyzed using Avisoft SASLab Pro software, which required the experimenter to manually assess each file to identify each vocalization and remove background noise, a very time‐consuming process. Mouse Song Analyzer has been developed in MATLAB as a fully automated program to analyze ultrasonic vocalizations. While Mouse Song Analyzer is more time‐efficient, a study compared the two analysis systems and found Mouse Song Analyzer to be less reliable than Avisoft.^30^ Avisoft was found to be superior to Mouse Song Analyzer detecting more ultrasonic vocalizations, especially when the total number of ultrasonic vocalizations was high. This liability would limit the use of Mouse Song Analyzer for automated seizure detection, where there are high numbers of vocalizations associated with GTCS.

Audio‐based sensors are being developed as noninvasive seizure detection devices for epilepsy patients, with several devices commercially available. The commercially available audio‐based devices are multimodal, detecting bed or respiration noises in addition to bed movement.^3^, ^31^, ^32^, ^33^ Ictal vocalizations are a common feature of generalized tonic–clonic and focal seizures in humans.^4^, ^7^ A recent retrospective Phase I study assessed the accuracy of identifying seizures in an epilepsy monitoring unit based on sound alone.^2^ The study showed that epileptologists were able to accurately identify hyperkinetic seizures and tonic–clonic seizures but failed to identify psychogenic seizures and seizures with nonmotor manifestations or automatisms only.^2^ Our results showing ictal vocalizations in *Scn1a* ^+/−^ mice during GTCS are consistent with the ictal cry common in epilepsy patients with generalized tonic–clonic or focal seizures. Dravet syndrome patients exhibit GTCS; however, it has not been reported whether ictal cries have been observed in this patient population. Future studies could explore whether ictal vocalizations are characteristic of seizures in Dravet syndrome patients and other preclinical seizure models. Audio‐based seizure detection could then become an important tool for evaluating seizures in models where the seizure semiology is subtle, such as absence seizures. A multimodal system where quantitative vocalization measures supplement behavioral and video‐EEG could have considerable diagnostic utility by improving confidence in seizure identification.

In summary, our study shows that ictal vocalizations occur in the *Scn1a* ^+/−^ mouse model of Dravet syndrome. Spontaneous seizures in *Scn1a* ^+/−^ mice were associated with a higher number of total vocalizations including both audible mouse squeaks and ultrasonic vocalizations. Audible mouse squeaks were primarily emitted during the preictal phase, while most ultrasonic vocalizations were detected during the ictal phase. The ultrasonic vocalizations during seizure were emitted at a higher frequency and duration than those emitted when a seizure was not present. Quantitative audio analysis could be further explored as an automated seizure detection tool for *Scn1a* ^+/−^ mice.

## AUTHOR CONTRIBUTIONS

LLA and JCA conceived the idea of the study. LLA, SPP, JLS, and JCA contributed to the design of the study. DEM, SPP, and LLA contributed to the acquisition and analysis of the data. LLA drafted the manuscript and all authors reviewed and approved the submitted version.

## CONFLICT OF INTEREST STATEMENT

None of the authors has any conflict of interest to disclose. We confirm that we have read the Journal's position on issues involved in ethical publication and affirm that this report is consistent with those guidelines.

## ETHICAL APPROVAL

We confirm that we have read the Journal's position on issues involved in ethical publication and affirm that this report is consistent with those guidelines.

## Supporting information


Figure S1–S2
