# Supplementary material for: Ictal vocalizations in the Scn1a +/− mouse model of Dravet syndrome
Source: Epilepsia Open. 2023 May 15;8(3):776–84. doi: 10.1002/epi4.12715 (PMC10472354; doi:10.1002/epi4.12715)
Supplement: Supplementary file 1 — Figure S1–S2 [file EPI4-8-776-s001.docx]

Supporting Information

**Ictal vocalizations in the *Scn1a^+/-^* mouse model of Dravet syndrome**

Lyndsey L. Anderson, Declan Everett-Morgan, Stela P. Petkova, Jill L. Silverman and Jonathon C. Arnold

**Supplementary Figure 1. Representative spectrograms of audible and ultrasonic vocalizations.** Representative spectrograms of audible mouse squeaks and ultrasonic vocalizations (bottom) from acoustic recordings of *Scn1a*^+/-^ mice during a generalized tonic-clonic seizure.


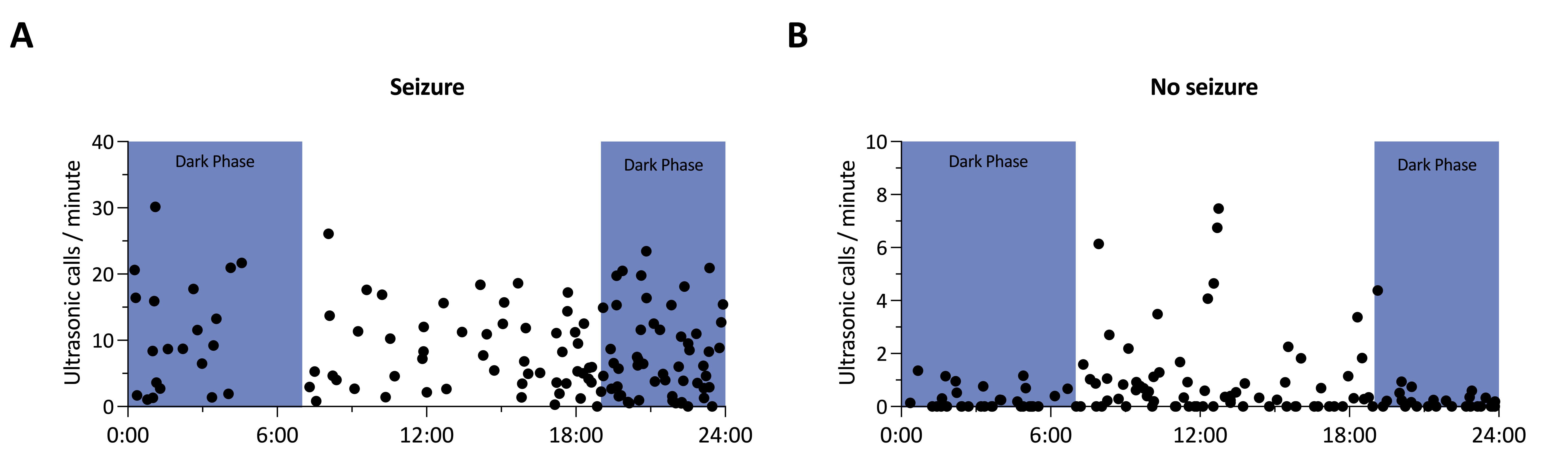


**Supplementary Figure 2. Ultrasonic vocalizations in *Scn1a*^+/-^ mice throughout the day.** Total number of ultrasonic vocalizations emitted in individual recordings of group-housed *Scn1a*^+/-^ mice. Ultrasonic vocalizations (> 20 kHz) across the day in acoustic recordings that (**A**) did and (**B**) did not contain a seizure. Note that the y-axis scale is different between graphs.
